# Pharmacist-Led Patient Education in Secondary Stroke Prevention: A Systematic Review

**DOI:** 10.21315/mjms-01-2025-043

**Published:** 2025-04-30

**Authors:** Daniek Viviandhari, Tri Murti Andayani, Paryono, Martina Sinta Kristanti

**Affiliations:** 1 Faculty of Pharmacy, Universitas Gadjah Mada, Yogyakarta, Indonesia; 2 Faculty of Pharmacy and Science, Universitas Muhammadiyah Prof. Dr. Hamka, Jakarta, Indonesia; 3 Faculty of Medicine, Public Health, and Nursing, Universitas Gadjah Mada, Yogyakarta, Indonesia

**Keywords:** patient education, pharmacist’s intervention, recurrent, secondary prevention, stroke

## Abstract

The recurrence rate of stroke, particularly ischaemic stroke, is considered high, which highlights the importance of secondary stroke prevention. Pharmacists are becoming increasingly involved in poststroke care. Ease of public access to community pharmacists provides opportunities for pharmacists to educate patients about the significance of secondary stroke prevention. The literature outlining comprehensive educational materials for stroke patients to prevent secondary stroke is still limited. This review aimed to obtain the details of the educational material presented by the pharmacist and to determine whether it led to positive outcomes—a comprehensive search involved PubMed, Scopus, Science Direct, and Cochrane Library databases. The Joanna Briggs Institute’s (JBI) critical appraisal techniques were utilised to analyse the quality assessment for all study categories. As many as 12 studies were deemed eligible for inclusion. There are seven main categories under which information on secondary stroke prevention education is presented: modifiable risk factors, medication therapy management (MTM), medication adherence, cardiovascular risk factors, stroke (disease state), patient engagement, and quality of life. Pharmacists, mostly in community settings, play an important role in managing stroke risk factors (blood pressure, low-density lipoprotein [LDL], and A1c), promoting medication adherence, preventing hospital readmissions, increasing the level of knowledge, and enhancing patient satisfaction, which showed a favourable effect on preventing stroke recurrence following the pharmacist’s educational intervention.

## Introduction

According to the Global Stroke Factsheet published in 2022, over 101 million people worldwide have had a stroke. Stroke is the leading cause of disability and ranks as the second leading cause of death globally. Over 12.2 million new strokes happen annually, and over the last 17 years, there has been a 50% increase in the lifetime chance of suffering a stroke. Besides, stroke is associated with significant health costs. Eighty-nine percent of disability-adjusted life years and 86% of stroke-related fatalities worldwide occur in lower- and lower-middle-income nations (1, 2).

A review study revealed that the recurrence rate of stroke, particularly ischaemic stroke, ranged from 5.7% to 51.3%. The rates of stroke recurrence do not change over time, even with the use of secondary prophylaxis medication (3). This emphasises the importance of secondary stroke prevention, which has been regarded for over 50 years as the cornerstone of stroke care. By controlling significant risk factors such as hypertension, dyslipidemia, diabetes, and smoking, optimal secondary preventive techniques can avert up to 80% of all recurrent strokes (4, 5).

Healthcare professionals should provide efficient interventions to monitor and control risk variables to prevent secondary stroke (6). Pharmacists are becoming increasingly involved in poststroke care (7). Many people find it comfortable to visit pharmacists for assistance regarding their health since they are easily accessible, particularly community pharmacists. This allows pharmacists to educate patients about the significance of secondary stroke prevention. Additionally, pharmacists work in clinics, hospitals, and other healthcare facilities, where they may save costs to the healthcare system while improving patient satisfaction, medication safety, health outcomes, and modifiable risk factors (8).

The literature outlining comprehensive educational materials for stroke patients to prevent secondary stroke is still limited. Pharmacists must have access to educational resources so they may adopt materials content and intervention strategies that they can then implement to improve services in their working areas: hospitals, outpatient clinics, or community settings. The impact of pharmacists on improving outcomes and their general involvement in primary and secondary stroke prevention were the main topics of previous systematic reviews (8, 9). The review also demonstrates how pharmacists play a variety of roles along the stroke treatment pathway and could improve several outcomes related to health (8). The content and results of the secondary stroke prevention education programme are the primary focus of this systematic review.

## Methods

The International Prospective Register of Systematic Reviews has the protocol for this study recorded under PROSPERO ID CRD42024500310.

### Literature Search

We performed a systematic and comprehensive literature search to identify all published studies relevant to the main topic using the following databases: PubMed, Scopus, Science Direct, and Cochrane Library. To finalise the data, more manual hand searches on Google Scholar were conducted. All searches were limited to clinical trial or randomised control trial (RCT) articles published between January 2003 and December 2023.

Three main themes were used in the search strategy: stroke, pharmacist intervention or education, and secondary stroke prevention. The following keywords were related to stroke (and MeSH terms): stroke, transient ischaemic attack, and ischaemic stroke. Keywords related to pharmacist intervention or education were as follows: pharmacist, pharmacists, community pharmacists, pharmaceutical care, pharmacist intervention, intervention, education, and pharmacist’s role. For secondary stroke prevention, the keywords included secondary prevention and secondary stroke prevention.

### Study Selection

Mendeley software was used to import and organise the search results. Duplicates were eliminated. With the title and abstract screening, one author (DV) independently found potentially relevant articles. When available, full texts of papers that might be of interest were then obtained. For eligibility, one author (DV) examined the full texts. When ambiguities were discovered, three authors (TMA, P, and MSK) evaluated full texts independently for eligibility. Discussion was used to settle disagreements. An article was considered relevant if pharmacists’ role was clearly defined in secondary stroke prevention management, educational intervention was delivered by pharmacists, and outcomes were included. Studies that dealt only with haemorrhagic strokes or those that were not written in English were excluded.

### Data Extraction

The primary author (DV) extracted the data using a data collection form, which was then verified by three additional researchers (TMA, P, and MSK). The information gathered consisted of the first author and year of publication, study population and number of samples, study aim(s), the design of the study and setting, explanation of pharmacist education, media for delivering education, duration of education, additional intervention, other healthcare professionals involved, primary outcomes measured, and secondary outcomes measured.

### Data Analysis

The results are presented in a descriptive and narrative synthesis manner because there was variability in the study design and outcome measurements.

### Quality Assessment

The included papers were categorised based on the study design to facilitate quality assessment. Every study design used critical appraisal tools from the Joanna Briggs Institute (JBI). All JBI checklist items received a response of either yes, no, uncertain/not clear, or not applicable (10). For every study included, the percentage of positive evaluations of the checklist items was determined to enable comparisons of the quality between the different study designs. Three categories were used to rank quality: less than 33% (low), 33–66% (medium), and above 66% (high) (11).

## Results

### Study Selection

**Figure 1:**
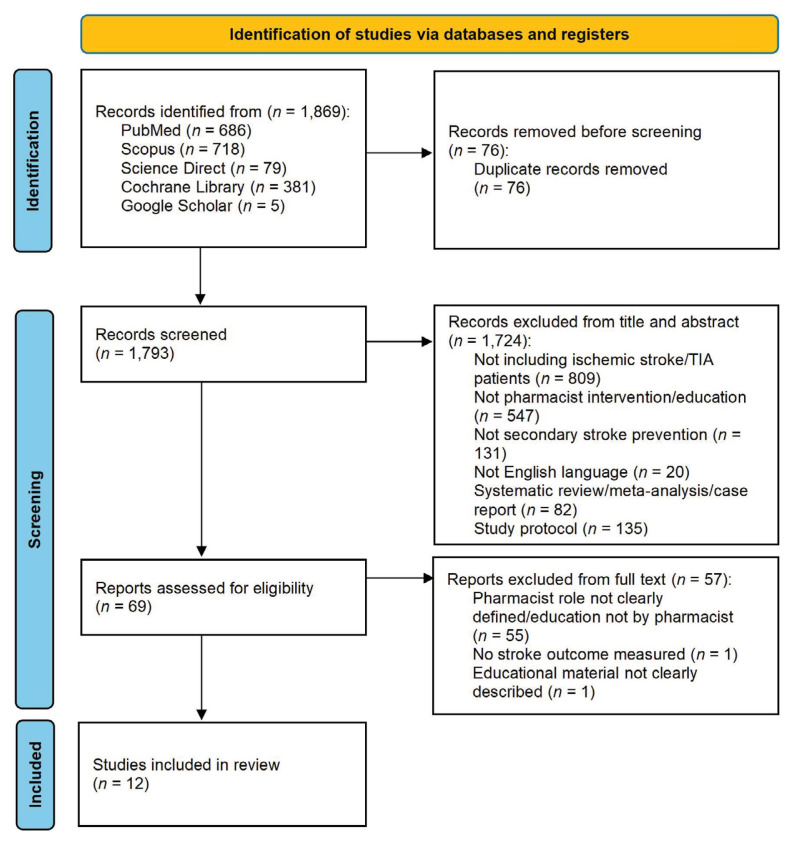
The screening procedure and exclusion criteria using the PRISMA flow chart

Five databases and a total of 1,869 articles were found through the literature search, and 1,793 items were found after the first search’s 76 duplicates were removed. During the title and abstract screening step, 1,724 papers were excluded, primarily because the research population did not include ischaemic stroke/transient ischaemic attack (TIA) patients (*n* = 809) or non-pharmacist intervention/education (*n* = 547). Sixty-nine papers were screened for full text, and an additional 57 articles were excluded due to the reasons indicated in Figure 1 and from the 12 included papers, all the data were extracted.

### Article Characteristics

The characteristics of the included studies are listed in Table 1. Six (or 50%) of these studies have been published in the last five years (12–17). The study population was entirely made up of poststroke/ischaemic stroke/TIA patients. Sample sizes ranged from 64 (18) to 455 (19) patients. In total, 2,618 patients were involved in this review. The results will likely be representative of the population increases with the sample size.

Based on Table 1, eight studies were controlled trials: four randomised by participants (12, 13, 20, 21); two stratified randomised (14, 22); and two non-randomised (17, 23). This non-randomised study was later categorised as a quasi-experimental study for quality assessment. Four studies were cohort studies: one prospective (18) and three retrospective (15, 16, 19) among the included researches. As much as 83.33% (*n* = 10) of the studies involved outpatients (in outpatient settings) (12–17, 19, 20, 22, 23). This indicates that the pharmacist’s role in secondary stroke prevention is mostly in community settings.

### Quality Assessment

Evaluating the quality of systematic reviews is justified by looking at confidence in the review findings. Quality assessment is an essential method for evaluating the overall strength of evidence about certain research topics. It is considered in contrast to the real effect of the intervention in the study process when assessing the methodological quality of a research design and implementation (24). Most of the research was of a high quality (75%, *n* = 9) (12–17, 19, 20, 23). As seen in Table 1, four out of six RCTs included in this review were considered high quality (12–14, 20). RCT scores ranged from 38.46% (medium quality) (22) to 84.62% (high quality) (13). Two quasi-experimental studies were both rated as high quality with a percentage of 88.89% (17) and 66.67% (23), while the included cohort studies are predominantly high quality (three out of four) (15, 16, 19).

### Description of Pharmacist’s Educational Intervention

**Figure 2:**
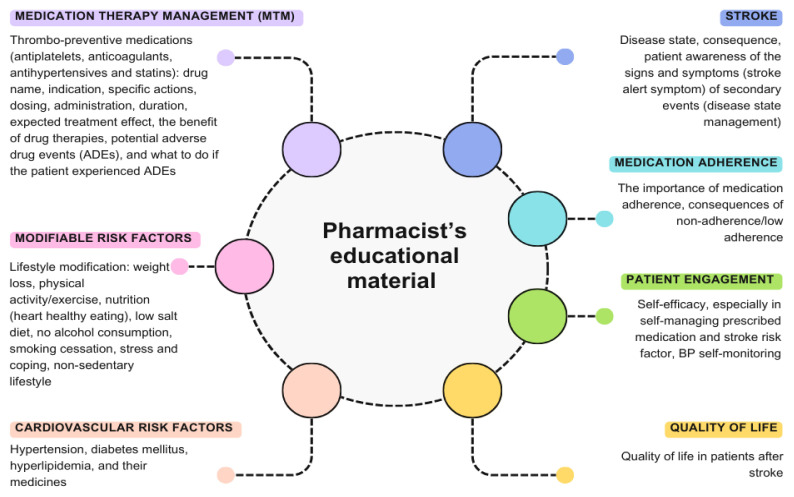
Pharmacist’s educational material

Table 2 summarises the pharmacist’s education. The educational material is described in detail in Figure 2. There were seven domains in the materials. The most discussed domain was modifiable risk factors (12, 14, 15, 17–22) (75%, *n* = 9), followed by medication therapy management (MTM) (12, 17, 21–23), medication adherence (12, 15, 17, 20, 22), and cardiovascular risk factors (14, 17, 18, 21, 23) (41.67%, *n* = 5, respectively). The least discussed topic was quality of life (17) (8.33%, *n* = 1).

All studies employed face-to-face media in providing/delivering educational interventions for patients, with various types such as standard face-to-face education (12, 15–20, 22, 23) (75%, *n* = 9), followed by face-to-face video viewing (13), face-to-face motivational interviews (21), and face-to-face group sessions (14) (8.33%, *n* = 1, respectively).

From the available data shown in Table 2 (not all studies include complete details of duration: either the duration of each pharmacist’s educational intervention session, the frequency of education, or patient follow-up over a certain period), the duration of each pharmacist’s educational intervention session was varied. One study states 15 minutes (17), two studies mention 20–30 minutes (12, 21), another two studies mention 60 minutes (14, 22), and one study takes 15–60 minutes (19). Another study explains that the duration of the educational session adapts to the inpatient’s clinical condition (18). The frequency of pharmacists delivering education varied from weekly (*n* = 2) (14, 19), monthly (*n* = 4) (12, 20, 22, 23), trimonthly (*n* = 2) (13, 23), and annually (*n* = 1) (19), to only once in the intervention period (*n* = 2) (16, 17). Patient follow-up ranged from three months (17), six months (12, 20, 22), and 11.2 months (18), to 12 months (13–15, 19, 21, 23).

Only 4 of 12 studies did not add interventions other than pharmacist education (13, 18, 19, 22). Most of the additional interventions were medication reviews (15, 16, 21, 23) (50%, *n* = 4). There are quite a variety of interventions other than medication reviews, such as medication consultation (12), risk factor evaluation, discussion with a physician, medication adjustment (20), telephone follow-up (21), rehabilitative exercise (14), care plan for community pharmacy from the clinical pharmacy at the hospital prior to discharge, DRPs identification and resolution (23), medication recommendation, reconciliation, and medication monitoring (15), risk factor modification (16), and educational leaflets (17) (Table 2).

To provide effective pharmaceutical care and ensure that patients receive the clinical results they need, pharmacists and other healthcare professionals must work well together. This is reflected in four studies that included the role of other health workers in the interventions carried out (13–15, 20). These other health workers are physicians, educationists, nurses, physiotherapists or kinesiologists, neurologists, and others involved in patient care (13–15, 20).

### Outcome Measures

An intervention’s effectiveness can be determined by assessing its measurable outcomes. Most studies in this review divide outcomes into primary and secondary. The most widely used primary outcome (found in four studies) is controlling stroke risk factors using the following parameters: blood pressure, LDL, and A1c (12–14, 22). This metric was utilised as a secondary endpoint in one study (19). According to four studies, the intervention group’s patient control of glucose (A1c), LDL, and blood pressure was noticeably superior to that of the control group (12–14, 19). Another trial failed on LDL and blood glucose parameters but showed promising benefits in blood pressure control alone (22). Three studies showed that the intervention positively increased medication adherence (12, 17, 18). Detailed outcomes are presented in Table 3.

As seen in Table 3, the appropriate parameter for describing the incidence of secondary stroke is hospital readmission (found in three studies [12, 16, 19]: two primary outcomes and two secondary outcomes) and either a 30-day readmission, readmission due to stroke/MI/PAD, or 90-day readmission. Only one study reported a negative impact on 30-day readmission due to intervention (16). Apart from this, all hospital readmission data showed a favourable outcome following the intervention (12, 16, 19).

The level of knowledge was primarily discussed in the three trials (13, 18, 21) as a secondary endpoint (13, 21), and each of the three demonstrated improved outcomes after the intervention was given. Patient satisfaction—including general satisfaction (18), satisfaction with the service from pharmacists (17, 21), and patient satisfaction with the tailored advice provided by pharmacists (23)—is an important additional parameter to assess, as reflected in four studies (17, 18, 21, 23), with positive results in all four studies.

## Discussion

An overview of the variety of educational interventions that pharmacists conduct on hospitalised patients and outpatients is provided in this review. These interventions are carried out either independently, by the pharmacist alone, or in conjunction with other medical specialists. Most studies provided similar results to a systematic review, which stated that pharmacists could participate in a stroke response team (9). Collaboration between pharmacists and other healthcare professionals in long-term stroke management is rarely discussed. A systematic review of interprofessional collaboration (IPC) in primary care (including the role of community pharmacists) found that IPC is useful in managing patients who are at risk of cardiovascular disease (25). The pharmacist can have a significant impact on the management of the stroke patient and the multidisciplinary stroke team during the rehabilitation phase. Nevertheless, creating efficient channels of communication with the various medical specialists on the stroke team is essential to the success of this input (26).

Interventions are either educational or in combination with other forms of intervention. To promote adherence to prescription drugs intended for cardiovascular disease (CVD) prevention, the Community Preventive Services Task Force suggests customised pharmacy-based interventions designed to assist patients in taking their prescription drugs as directed. Among the interventions are the following: assessment and tailored guidance and services (a pharmacist develops and provides individualised advice and services to eliminate or minimise obstacles based on the findings of the patient’s assessment). Personalised guidance can take the form of motivational interviewing sessions or targeted pharmacological counselling. A few examples of customised services include synchronising medicine refills, providing better follow-up, and providing patient resources such as pillboxes, medication cards, and calendars (27).

Pharmacy education materials on secondary stroke prevention vary from general to specialised topics. Given that community pharmacists are the healthcare professionals with whom the general population interacts most readily and regularly, the topic of community pharmacists’ roles is highly relevant. The evidence on managing hypertension and dyslipidemia lends support to the inclusion of a pharmacist in an interdisciplinary secondary stroke prevention clinic. Pharmacy professionals are uniquely positioned to identify, treat, and prevent medication-related issues, besides providing ambulatory stroke survivors with pharmaceutical treatment (28). Pharmacists could raise public awareness of the need to manage modifiable risk factors to prevent stroke recurrence.

### Pharmacist’s Educational Material

Modifiable risk factors have become the most common material presented to stroke patients in many studies. Modifiable risk factors must be identified to reduce stroke prevalence in the general population, and risk reduction measures must demonstrate their efficacy (29). An observational study indicated that a significant public health campaign should start with an emphasis on educating the public about the risk factors for stroke and the required interventions (30). According to a case-control study, 10 risk factors are associated with 90% of the risk of stroke, three of which are hypertension, diabetes mellitus, and current smoking. The risk of having a stroke can be significantly decreased with targeted therapies that lower blood pressure and encourage people to quit smoking, engage in physical activity, and consume a balanced diet (31).

MTM was one of the second most discussed topics. Antihypertensive therapy, glycemic control, cholesterol-lowering therapy, and antithrombotic therapy are the cornerstones of medical treatment to prevent secondary stroke (32). The best combination of secondary preventive drug classes is linked to a markedly decreased risk of major vascular events, stroke, and mortality (33). MTM provided by pharmacists enhanced the clinical results of patients with dyslipidemia, hypertension, or diabetes (all three of which are strong risk factors for stroke/recurrent stroke), according to a systematic review of MTM interventions (34). Based on a study, pharmacists who participate in outpatient MTMs are better able to identify drug-related problems (DRPs) and promptly create personalised medication-related action plans for patients (35).

Even though patient engagement is the second least discussed, its effect on increasing patient awareness is important in supporting the clinical outcome of patient therapy. According to one study, patient involvement in stroke rehabilitation decisions is crucial since it seems to be linked to addressing patients’ demands for health services in six areas of difficulty: falls, exhaustion, emotions, memory, speaking, and reading (36).

### Outcome

In line with the educational materials presented in this review, five major outcomes were reviewed. In general, this review offers promising data supporting the implementation of pharmacist education in preventing secondary stroke. Only 4 of 12 studies showed a negative impact on the primary outcome. Pharmacist intervention showed an ability to manage the risk factors well, make the patients adhere to treatment, prevent hospital readmission, increase the level of knowledge, and increase patient satisfaction.

### Limitations

This review has some limitations. One is that only a few studies have examined the role pharmacists can play in secondary stroke prevention through education. Another is that because the study design and outcome measurements varied, the intervention’s results were not always consistent.

## Conclusion

The content of material presented in secondary stroke prevention education is divided into seven primary domains: modifiable risk factors, MTM, medication adherence, cardiovascular risk factors, stroke (disease state), patient engagement, and quality of life. Pharmacists, mostly in community settings, play an important role in managing stroke risk factors (blood pressure, LDL, and A1c), promoting medication adherence, preventing hospital readmissions, increasing the level of knowledge, and enhancing patient satisfaction. These interventions showed a favourable effect in preventing stroke recurrence.

## Figures and Tables

**Table 1:** Studies characteristics and quality assessment

No.	First author and year of publication	Study population and number of samples	Study aim(s)	Study design and setting	JBI quality (%)	JBI category
1.	Wang et al. (2021) (12)	Patients admitted to the hospital’s neurology ward following an ischaemic stroke; 166 patients (84 intervention, 82 control)	To assess how a pharmaceutical care program affects hospital readmissions and risk factor control (blood pressure, blood sugar, lipid profile, and medication adherence) in the context of poststroke treatment	Randomised controlled trial; outpatient setting	69.23	High
2.	Appalasamy et al. (2020) (13)	Outpatient at Neurology Clinic; 216 patients (108 intervention, 108 control)	To investigate the impact of video narratives integrated with Health Belief frameworks on patients with stroke in a local environment regarding their MUSE and related aspects	Randomised controlled trial; outpatient setting	84.62	High
3.	McAlister et al. (2014) (20)	Adult residents of the community who have a minor or no disability following an ischaemic stroke or TIA; 275 patients (139 pharmacist intervention, 136 nurse intervention)	To assess the effect on global vascular risk of two forms of case management—with a pharmacist or with a nurse—added to standard treatment	Randomised controlled trial; outpatient setting	69.23	High
4.	Hedegaard et al. (2014) (21)	TIA and stroke patients; 203 patients (102 intervention, 101 control)	To determine whether a comprehensive intervention, such as MI, is beneficial in enhancing medication adherence for the prevention of secondary strokes	Randomised controlled trial; hospital setting	53.85	Medium
5.	MacKay-Lyons et al. (2022) (14)	Patients with a first probable or confirmed TIA or NDS within three months; 196 patients (98 intervention, 98 control)	To examine the effects of short- and long-term community-based education programmes and rehabilitative exercise in addition to medication management to lower risk factors of vascular in individuals who have had NDS or TIA	Randomised controlled trial (stratified by stroke prognosis or death within two years); outpatient setting	69.23	High
6.	Chiu et al. (2008) (22)	Outpatients with ischaemic stroke who had been going to hospital clinics regularly for over a year after their stroke; 160 patients (80 intervention, 80 control)	To assess the effectiveness of pharmacist intervention at a tertiary referral hospital and the suitability of managing MRF in a group of outpatients with ischaemic stroke	Randomised controlled trial (stratified by age and sex); outpatient setting	38.46	Medium
7.	Firat et al. (2023) (17)	Patients who suffered their first stroke and were admitted to the hospital; 98 patients (48 intervention, 50 control)	To ascertain the effect of clinical pharmacist education in the three months following hospital discharge on quality of life and medication adherence in patients who had suffered their first stroke	Non-randomised controlled trial; outpatient setting	88.89	High
8.	Hohmann et al. (2010) (23)	Individuals who had an ischaemic stroke or TIA and had more than 30 points of Barthel index at hospital discharge and living at home; 255 patients (90 intervention, 165 control)	To enhance patients’ HQL following an ischaemic stroke or TIA; ensure an efficient secondary prevention; and raise patients’ satisfaction with pharmacists’ prescription recommendations	Non-randomised controlled trial; outpatient setting	66.67	High
9.	Ben Nasr et al. (2018) (18)	Hypertensive individuals who were hospitalised at the stroke centre following a TIA or an acute episode of haemorrhagic or ischaemic stroke; 64 patients	To assess how an educational programme affects hypertensive stroke patients’ knowledge and blood pressure control	Prospective cohort study; inpatient setting	36.36	Medium
10.	Greger et al. (2021) (15)	Patients treated after a stroke or TIA were assessed by an outpatient neurology practice; 342 patients (171 intervention, 171 control)	To ascertain if, in individuals at risk of recurrent stroke or TIA, compared to standard-of-care alone, pharmacist medication intervention plus whole blood aggregometry anti-platelet medication monitoring improved anti-platelet treatment response	Retrospective cohort study (a single-centre, retrospective chart review); outpatient setting	81.81	High
11.	Nathans et al. (2020) (16)	Adult patients who were sent to their homes after receiving an initial diagnosis of stroke or TIA and who visited a PSTCC; 188 patients (94 intervention, 94 control)	To offer risk factor adjustment to lower the chance of recurrence and hospital readmissions by establishing a poststroke transition of care clinic run by pharmacists	Retrospective matched-cohort study; outpatient setting	72.73	High
12.	Andres et al. (2019) (19)	Patients who had suffered a TIA or stroke following their discharge from the hospital were referred to the SPC; 455 patients (257 intervention, 198 control)	To ascertain whether patients who receive SPC care fare better than patients with standard care	Retrospective cohort study; outpatient setting	81.81	High

Notes: HQL = health-related quality of life; MI = motivational interviewing; MRF = modifiable risk factors; MUSE = medication understanding and use self-efficacy; NDS = non-disabling stroke; PSTCC = poststroke transitions of care clinic; SPC = pharmacist-run stroke prevention clinic; TIA = transient ischaemic attack

**Table 2:** Description of pharmacist’s education of included studies

No.	First author and year of publication	Description of pharmacist education	Media for delivering education, duration of education	Additional intervention	Other healthcare professionals involved
1.	Wang et al. (2021) (12)	The focus of secondary prevention of ischaemic stroke education was on MTM, lifestyle adjustments, and disease status management. During MTM consultations, the following topics were covered: drug name, indication, the benefit of medication therapies, dosage, administration, duration, projected therapeutic outcome possible adverse drug reactions (ADEs), and what to do if the patient experienced ADEs. Self-monitoring of blood pressure and blood sugar, as well as the need for medication adherence were highlighted in the education on disease state management	Face-to-face pharmacy consultations; monthly pharmaceutical care (seven times): at ward discharge (around 30 min), a 1-month clinic visit (requiring follow-up assessment from a physician), four phone calls or WeChat^®^ follow-ups (lasting roughly 20 min each), and the 6-month clinic visit (evaluating study parameters), follow-up for six months	Medication consultation from a dispensing pharmacist for refills, which occur at the hospital outpatient pharmacy every 30 days	-
2.	Appalasamy et al. (2020) (13)	The motivational messaging emphasised self-efficacy, particularly about stroke risk factors and self-managing prescribed medicines. To enhance the engagement elements of the statements, real neurologists and stroke survivors depicted their experiences with genuine emotions	Face-to-face video narratives; intervention was delivered three times (baseline, three months, six months), follow-up for 12 months	-	Doctors, educationists
3.	McAlister et al. (2014) (20)	Education on medication adherence and lifestyle advice, such as exercise, a low-sodium diet, and quitting smoking	Face-to-face education; monthly visits, follow-up for six months	The pharmacist performed a serial risk factor evaluation, shared the results with primary care physicians, and started and adjusted antihypertensive and/or cholesterol-lowering medication as necessary	Primary care physicians, nurse
4.	Hedegaard et al. (2014) (21)	Education on secondary prevention, hypertension, stroke, risk factors, thrombopreventive drugs (statins, antiplatelets, anticoagulants, and antihypertensives), medication adherence, and lifestyle modification	Face-to-face MI; 20–30 minutes intervention, follow-up for 12 months	Review of medications and phone follow-up by a pharmacist	-
5.	MacKay-Lyons et al. (2022) (14)	1. Heart-healthy diet: the fundamentals; 2. Establishing goals; 3. Workout: Fundamentals; 4. Blood pressure self-monitoring and cardiovascular risk factors; 5. Nutrition: expanding on the fundamentals; 6. Exercise: Expanding upon the fundamentals; 7. Drugs for cardiovascular disease; 8. A healthy weight; 9. Giving up smoking; 10. Coping and stress; and 11. Adjusting a healthy diet	An interactive group session for participants, family members, and/or caregivers was led by a multidisciplinary team; 60 minutes, once weekly, three months, 6- and 12-month follow-up	Rehabilitative exercise	Trained physiotherapists or kinesiologists on exercise. Multidisciplinary team on education session
6.	Chiu et al. (2008) (22)	Education on the effects of drugs, lifestyle modification, treatment objectives, advantages of treatments, the importance of adherence, drug interaction verification, and informing patients of side effects	Face-to-face education; monthly 1-hour intervention, follow-up for six months	-	-
7.	Firat et al. (2023) (17)	Education on stroke (definition, prevalence, controlling stroke risk factors, potential aftereffects of stroke), information about the prescribed drugs and potential side effects, the significance of medication adherence, the results of poor or non-adherence, and quality of life	Face-to-face education; 15-minute intervention, follow-up for one and three months	Educational leaflets given at the end of the educational interview	-
8.	Hohmann et al. (2010) (23)	Education on medications, particularly prevention of secondary stroke associated with the mechanism of actions, adverse effects, and drug-drug-interactions, and also medications used to treat cardiovascular risk factors like diabetes, hypertension, and hyperlipidemia	Face-to-face education; once a month, at least every three months, follow-up for 12 months	The hospital’s clinical pharmacist’s care strategy for the community pharmacy prior to discharge, medication review, and identification and resolution of DRPs	-
9.	Ben Nasr et al. (2018) (18)	Education on self-measurement of blood pressure, changes in lifestyle with an emphasis on nutrition, exercise, and the physiopathology of stroke, in addition to risk factors, symptoms, outcomes, and therapy for hypertension	Face-to-face education; patients were presented with the education programme two until three days after being admitted, and the duration of the sessions was adjusted based on their condition and level of exhaustion. The average follow-up period was 4.9 months following the educational session	-	-
10.	Greger et al. (2021) (15)	Education on medication adherence, recurrent stroke’s key modifiable risk factors, such as smoking cessation and the use of hormone replacement therapy	Face-to-face education; follow-up for 12 months	Medication recommendation (dosage adjustments, formulation modifications, switching to a different medicine, drug interactions), medication review and reconciliation, anti-platelet medication monitoring with WBA	The neurologist and/or other healthcare professionals involved
11.	Nathans et al. (2020) (16)	Education on disease state, patient knowledge of the warning signs and symptoms (stroke alert symptoms) of secondary occurrences	Face-to-face education; 1-time visit	Medication management (dosage adjustments, therapy additions or withdrawals, ordering and tracking laboratory testing), risk factor modification, and, if necessary, acting as a conduit to primary care services	-
12.	Andres et al. (2019) (19)	Education on nutrition, physical activity, alcohol intake, body weight, and illicit substance use	Face-to-face education; 15-minute to 1-hour interventions, follow-up weekly to annually	-	-

Notes: ADEs = adverse drug events; DRPs = drug-related problems; MTM = medication therapy management; WBA = whole blood aggregometry

**Table 3:** Outcomes of included studies

No.	First author and year of publication	Primary outcomes measured	Secondary outcomes measured
1.	Wang et al. (2021) (12)	Concerning antihypertensive medications (*P* = 0.031), anti-diabetic medications (*P* = 0.02), and lipid-lowering medications (*P* = 0.022), medication adherence rates increased significantly in the IG. The goal surrogate risk factor control indicators of LDL-C (*P* = 0.02) and haemoglobin A1c (*P* = 0.038) were attained by more patients in IG	Readmissions to the hospital occurred in IG at a significantly lower rate than in CG (*P* = 0.03)
2.	Appalasamy et al. (2020) (13)	In the intervention group, there were significant differences both within and between groups according to MUSE mean score differences for antithrombotic, antihypertensive, and all drug categories (*P* < 0.05). In comparison to the CG, continuous BP monitoring was significantly higher (*P* = 0.023). Significant mean differences in BP control over time were shown by repeated measure analysis between the IG and CG (*P* < 0.001)	The beneficial effects of the intervention were also evident in comparable patterns for knowledge, perceptions of illness, and belief about medication
3.	McAlister et al. (2014) (20)	There were reductions in the absolute global vascular risk estimates for both study arms: on the FRS, *P* = 0.44 between arms; on the CDLEM, *P* = 0.37. These decreases continued after a year: *P* = 0.20 on the CDLEM and *P* = 0.83 for the FRS	-
4.	Hedegaard et al. (2014) (21)	The intervention group had an MPR of 0.95, whereas the control group had a median MPR of 0.91. Of the patients, 28 and 21%, respectively, were nonadherent (MPR < 0.80). Over time, the median MPR dropped in both groups. No statistically significant difference between the groups according to comparisons	Regarding the clinical outcome or adherence and persistence to particular thrombopreventive medications, no significant variations were observed. Patients in IG expressed satisfaction with the service; almost 50% of respondents said their knowledge about medications rose, and 33% reported feeling more confident when using them
5.	MacKay-Lyons et al. (2022) (14)	DBPrest (*P* = 0.04) and LDL-C (*P* = 0.02) showed significant between-group differences at post-intervention that favoured the PREVENT group over the UC. Several metrics demonstrated improving trends in the PREVENT group from the baseline to the 6-month follow-up but not at the 12-month follow-up	-
6.	Chiu et al. (2008) (22)	Before the trial, the percentages of the control and intervention groups with acceptable blood pressure, lipid, and glucose control were not significantly different. A significant difference in BP control was found at the end of the trial	-
7.	Firat et al. (2023) (17)	A significant increase in treatment adherence during the research period (*P* < 0.001) was found in IG. IG experienced significantly larger changes in treatment adherence than CG did from the day of discharge to the first month and the day of discharge to the third month (*P* < 0.001). On the other hand, there were no appreciable variations in the level of treatment adherence between the first and third months (*P* > 0.005)	Each group’s overall SSQOL score increased significantly (*P* < 0.001), but no discernible differences between the groups were observed during the trial. Patients in IG had higher PSPSQ scores than patients in CG on three sub-dimensions (quality of treatment, pharmacist-patient interaction, and overall satisfaction) (*P* < 0.001)
8.	Hohmann et al. (2010) (23)	During the study, there was no discernible change in HQL among the patients in the IG. A statistically significant decrease was observed in the HQL for the CG in 7/8 subscales and both SF-36 summary measures	Patients in the IG were noticeably more satisfied with the tailored medication advice provided by the pharmacists
9.	Ben Nasr et al. (2018) (18)	From 77.9 to 94.1%, the correct response rate rose, and from 52.9 to 80.8%, the sure response rate grew. Patients reported self-measurement of their blood pressure more frequently and with better adherence to their medications. They were highly satisfied. Knowledge evolution and baseline knowledge were found to be negatively correlated	-
10.	Greger et al. (2021) (15)	Following pharmacist intervention and PFT, 83% of patients were deemed responsive to their anti-platelet medication, up from 27% in the IG at baseline (*P* < 0.0001)	-
11.	Nathans et al. (2020) (16)	The 30-day readmission rates did not show any change (difference *P* = 0.001).	Ninety-day readmissions showed a significant There was no discernible difference in the 30- or 90-day incidence of stroke recurrence or ER visits
12.	Andres et al. (2019) (19)	The SPC group exhibited a statistically significant reduction in stroke/TIA, myocardial infarction, and new or incidental PAD readmissions as compared to the CG (*P* = 0.013)	In the SPC group, there was an improvement in low-density lipoprotein, haemoglobin A1c, blood pressure, and smoking status

Notes: BP = blood pressure; CDLEM = cardiovascular disease life expectancy model; CG = control group; DBPrest = resting diastolic blood pressure; FRS = Framingham Risk Score; haemoglobin A1c = glycated/glycosylated haemoglobin; HQL = health-related quality of life; IG = intervention group; LDL-C = low-density lipoprotein-cholesterol; MPR = median medication possession ratio; MUSE = medication understanding and use self-efficacy; PAD = peripheral arterial disease; PFT = platelet function testing; PREVENT = programme of rehabilitative exercise and education to avert vascular events after non-disabling stroke or transient ischaemic attack; PSPSQ = patient satisfaction with pharmacist services questionnaire; SF-36 = short form-36 questionnaire; SPC = pharmacist-run stroke prevention clinic; SSQOL = stroke-specific quality of life scale; TIA = transient ischaemic attack; UC = usual care
